# Program evaluation of an ORS and zinc scale-up program in 8 Nigerian states

**DOI:** 10.7189/jogh.09.010502

**Published:** 2019-06

**Authors:** Felix Lam, Ahmad Abdulwahab, Jason Houdek, Olajumoke Adekeye, Mohammed Abubakar, Adewale Akinjeji, Tiwadayo Braimoh, Obinna Ajeroh, Melinda Stanley, Nancy Goh, Kate Schroder, Owens Wiwa, Nnenna Ihebuzor, Marta Rose Prescott

**Affiliations:** 1 Clinton Health Access Initiative, Boston, Massachusetts, USA; 2 Clinton Health Access Initiative, Abuja, Nigeria; 3 National Primary Health Care Development Agency, Abuja, Nigeria; † This paper is dedicated to Dr Nnenna Ihebuzor who passed away on March 24, 2018. The study and the program described would not have been possible without her tireless efforts.

## Abstract

**Background:**

In Nigeria, diarrhea is the second leading killer of children under five. Between 2012-2017, the Clinton Health Access Initiative, Inc. (CHAI) and the Government of Nigeria implemented a comprehensive program in eight states aimed at increasing the percentage of children under five with diarrhea who were treated with zinc and oral rehydration solution (ORS). The program addressed demand, supply, and policy barriers to ORS and zinc uptake through interventions in both public and private sectors. The interventions included: (1) policy revision and partner coordination; (2) market shaping to improve availability of affordable, high-quality ORS and zinc; (3) provider training and mentoring; and (4) caregiver demand generation.

**Methods:**

We conducted cross–sectional household surveys in program states at baseline, midline, and endline and constructed logistic regression models with generalized estimating equations to assess changes in ORS and zinc treatment during the program period.

**Results:**

In descriptive analysis, we found 38% (95% CI = 34%-42%) received ORS at baseline and 4% (95% CI = 3%-5%) received both ORS and zinc. At endline, we found 55% (95% CI = 51%-58%) received ORS and 30% (95% CI = 27%-33%) received both ORS and zinc. Adjusting for other covariates, the odds of diarrhea being treated with ORS were 1.88 (95% CI = 1.46, 2.43) times greater at endline. The odds of diarrhea being treated with ORS and zinc combined were 15.14 (95% CI = 9.82, 23.34) times greater at endline. When we include the interaction term to investigate whether the odds ratios between the endline and baseline survey were modified by source of care, we found statistically significant results among diarrhea episodes that sought care in the public and private sector. Among cases that sought care in the public sector, the predictive probability of treatment with ORS increased from 57% (95% CI = 50%-65%) to 83% (95% CI = 79%-87%). Among cases that sought care in the private sector, the predictive probability increased from 41% (95% CI = 34%-48%) to 58% (95% CI = 54%-63%).

**Conclusions:**

Use of ORS and combined ORS and zinc for treatment of diarrhea significantly increased in program states during the program period.

In Nigeria, diarrhea is the second leading killer of children under the age of five. In 2011, nearly 90 000 children under five died from diarrhea [1]. Oral rehydration solution (ORS) can prevent up to 93% of diarrheal deaths and zinc can reduce the duration of diarrhea, particularly in malnourished children. [2,3]. In 2004, the World Health Organization (WHO) and United Nations Children’s Fund (UNICEF) issued a joint statement endorsing the combined treatment for acute diarrhea [4]. The Federal Ministry of Health of Nigeria adopted this policy in 2010 and, in 2012, the Federal Ministry of Health (FMOH) and National Primary Health Care Development Agency (NPHCDA) launched the *Essential Childhood Medicines Scale-up Plan: 2012-2015,* the country’s first ever national roadmap for reducing child mortality by increasing access to life-saving treatments—including zinc and ORS [5]. Between 2013 and 2017, the Clinton Health Access Initiative, Inc. (CHAI), with funding from the Norwegian Agency for Development Cooperation (Norad) and Global Affairs Canada (GAC), supported the Government of Nigeria to implement a program that aligned with the strategies and activities under the *Essential Childhood Medicines Scale-up Plan*. This program included interventions to ensure widespread availability and affordability of optimal ORS and zinc products, strengthen diagnosis and treatment practices of public and private providers, and generate demand amongst caregivers of children. The program was focused in eight states – Bauchi, Cross River, Lagos, Kaduna, Kano, Katsina, Niger, and Rivers [6].

## Program description

The program approach aimed to address demand, supply, and policy barriers to ORS and zinc uptake in both public and private sectors. The interventions were categorized into four major areas: (1) policy revision and partner coordination; (2) market shaping to improve availability of affordable, high-quality ORS and zinc; (3) provider training and mentoring; and (4) caregiver demand generation. In Table S1 in **Online Supplementary Document**, we describe in detail the program activities under each of these four areas roughly separated across two program phases: between the baseline and midline surveys and between the midline and endline surveys.

For policy revision and partner coordination, the program partnered with the FMOH, NPHCDA, and state Ministries of Health to establish coordination mechanisms at national and state levels. Led by the government, the National Essential Medicines Coordinating Mechanism (NEMCM) consisted of various government departments, private industry, and non-governmental partner organizations [7]. The aim of the NEMCM was to mobilize resources for implementation of Nigeria’s *Essential Childhood Medicines Scale-up Plan* and to align and coordinate key stakeholders around the scale-up plan and updates to national treatment policies. By 2014, the NEMCM partners supported government efforts to revise to the national treatment guideline to recommend ORS and zinc for childhood diarrhea, add zinc to the national and state Essential Medicines Lists (EML), and broadly communicated the policy allowing zinc to be available over-the-counter. The NEMCM also conducted a partner mapping exercise to identify investments by non-governmental stakeholders and coordinated those investments to expand the reach of program implementation to reach underserved areas. For example, the Sustaining Health Outcomes through the Private Sector (SHOPS) program selected Benue and Kebbi state for additional investments in ORS and zinc scale-up based on the partner mapping exercise [8].

For market shaping, the program engaged local manufacturers to encourage investments in production, promotion, and sales of optimal zinc and ORS products— including zinc dispersible tablets (DT), the WHO-recommended low-osmolarity ORS formulation, and co-packaged ORS and zinc. Market intelligence on Nigeria’s demand and planned orders was provided to suppliers through regular forums and reports. In addition, suppliers received one-on-one technical assistance on product registration, cost reduction, marketing, and product and packaging optimization strategies. This support led to the introduction of four new low-osmolarity ORS, six zinc DT, and seven co-packaged ORS and zinc products into the Nigerian market. Beyond the technical support to local suppliers, the program worked with Nigeria’s National Agency for Food and Drug Administration and Control (NAFDAC) to encourage suppliers to switch to a low-osmolarity ORS formulation. Prior to the program, 21 different ORS products were already available in the Nigerian market, though there was only one low-osmolarity ORS registered in the country, no zinc DT, and no co-packs. By the end of the program, there were 35 low-osmolarity ORS, 12 zinc DT, and 10 co-packs registered with NAFDAC. **Table 1** presents the number of low-osmolarity ORS, zinc DT, and co-packs registered with NAFDAC prior to the program and by program’s end. While the program partnered directly with a handful of willing suppliers to strengthen the local supply base of ORS and zinc, the program was overall agnostic to the brand of ORS and zinc used by patients and aimed to increase the overall use of ORS and zinc by patients.

**Table 1:** Number of ORS and zinc products registered in Nigeria*

Product type	Prior to program (2012)	By end of program (2016)
L-ORS (All ORS formulations)	1 (21)	35 (58)
Zinc dispersible tablets	0	12
Co-packaged ORS and zinc	0	10

L-ORS – low-osmolarity oral rehydration solution, ORS – oral rehydration solution

*Source: NAFDAC registration database, 2012-2016.

Furthermore, the program worked along the entire private-sector supply chain in Nigeria to expand the reach of ORS and zinc in rural areas. The program used innovative private sector strategies and streamlined distribution models targeting wholesalers, sub-distributors, and retailers. For example, the program placed brand-agnostic marketers at wholesale outlets to distribute educational materials and encourage private retailers patronizing wholesale outlets to stock ORS and zinc. The market shaping activities in the private sector led to a large increase in ORS and zinc sales by major ORS and zinc suppliers in Nigeria. **Table 2** presents annual sales of ORS, zinc, and co-packs from seven major local suppliers during the program period.

**Table 2:** Procurement and sales volumes of ORS and zinc by year

Product type	State government procurement			Private supplier sales (7 suppliers)		
**2014**	**2015**	**2016**	**2014**	**2015**	**2016**
ORS	1 167 733	245 200	196 500	7 111 308	17 015 062	27 165 336
Zinc	131 997	33 245	245 550	2 116 897	4 989 751	7 070 724
Co-packs	58 832	383 053	427 015	855 449	3 561 002	8 603 096

ORS – oral rehydration solution

The program also supported the state governments to optimize planning and procurement of ORS and zinc, including introduction of an ORS and zinc co-pack, into public facilities. The program provided technical assistance to quantify ORS and zinc demand in public facilities and advocated for inclusion of these commodities in the state procurement budgets. Additionally, the program worked with state drug management agencies to include ORS and zinc in the state drug revolving funds (DRFs) and negotiated reduced prices. By the end of the program, we observed an increasing preference by state governments for procuring co-packs. As shown in **Table 2**, public sector procurement gradually shifted from single ORS and zinc to co-packs.

The program aimed to switch the practices of health care providers to use ORS and zinc for treatment of pediatric diarrhea through multiple interventions. The program implemented targeted activities in the public and private sector. First the program conducted diarrhea management trainings with leaders of health worker professional groups, such as the Nigerian Medical Association (NMA), Pharmaceutical Society of Nigeria (PSN), National Association of Nigerian Nurses and Midwives (NANNM), and National Association of Proprietary Patent Medicine Vendors (NAPPMED) to secure buy-in. The program subsequently rolled out state-level trainings to nearly 20 000 health care providers in the public sector. In the private sector, the program partnered with the NAPPMED and the Pharmacists Council of Nigeria (PCN) to conduct state-level trainings to nearly 19 000 patent and proprietary medicine vendors (PPMVs). Following up on the state-level trainings, the program worked with existing government supervisory structures to provide individual mentorship of public health care workers on diarrhea case management. Similarly, in the private sector, the program worked with NAPPMED and PCN to conduct shop-to-shop detailing with their network of PPMVs and pharmacists.

The fourth area of intervention was to directly reach caregivers and encourage them to seek care early and provide them with education on ORS and zinc. The program worked with key influencers in the community, such as religious leaders, female vanguard associations, Islamiyah schools, and local health care workers, to incorporate messages in their communication platforms. A radio campaign was also implemented in selected states.

The first half of the program focused on increasing procurement of ORS and zinc in the public sector and getting negotiated prices, mapping private sector wholesale and retail networks and implementing activities to push ORS and zinc through the private sector supply chains, and large-scale training programs for both private and public sector providers to orient them to ORS and zinc and link them to suppliers. Limited community engagement occurred during the first half of the program. In the second half of the program, the focus switched to conducting multi-contact interventions that leveraged sustainable channels, such as government supportive supervision activities and pharmaceutical detailing. The majority of community engagement activities also occurred in the second half of the program, such as utilizing key influencers, facility-based “health talks”, and radio.

## Study objectives

The purpose of this study is to evaluate the program and to present the results of the household surveys conducted at baseline, midline, and endline using analytical techniques. To estimate the potential program impact, we focused on five outcomes: 1) the change over time in care-seeking for children with diarrhea in the past two weeks; 2) the change over time in treatment with ORS for children with diarrhea in the past two weeks; 3) the change over time in treatment with combined ORS and zinc; 4) whether the change over time in treatment with ORS was modified by the source of care (ie, home, public sector, private sector, other sector, or multiple sectors); and 5) whether the change over time in treatment with combined ORS and zinc was modified by the source of care.

## METHODS

### Evaluation study design and setting

**Figure 1:**
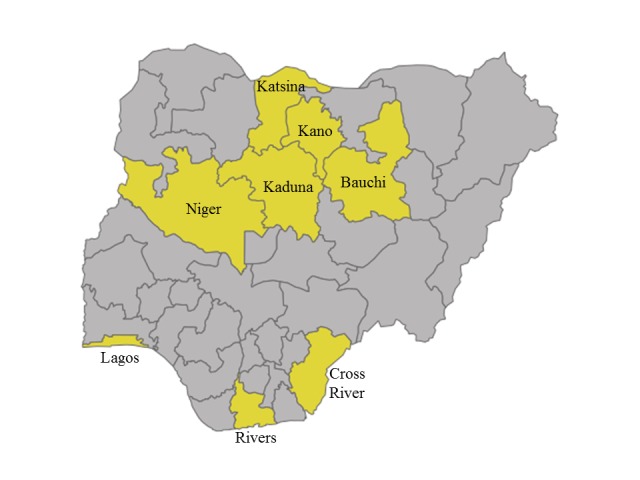
Map of program states.

This evaluation consisted of repeated cross-sectional, multi-stage, cluster-based household surveys carried out separately in Bauchi, Cross River, Kaduna, Kano, Katsina, Lagos, Niger, and Rivers (**Figure 1**). The eight states represent a wide variety of cultures, health, and economic outcomes and cover five out of the six geopolitical zones (only South East is not represented). According to the 2006 census, out of the 36 states and the Federal Capital Territory of Abuja, these 8 states combined represent 36% of the under-five population and 34% of the total population [9]. The state-level under-five mortality varies and estimates ranged from the highest in Bauchi state (186 per 1000 live births) to the lowest in Lagos and Rivers (74 and 96 per 1000 live births) [10]. Across all states, diarrhea is a leading cause of deaths among children under five, and the eight states represent 40% of the total pediatric diarrhea burden in Nigeria. Prior to the program, the UNICEF MICS 2011 survey estimated ORS coverage to be the lowest in Bauchi (12%) and highest in Lagos (62%) [11].

### Study population

The study population was children under five who resided in one of our eight states within Nigeria where the program was implemented and had diarrhea in the past two weeks.

### Sampling design

**Figure 2:**
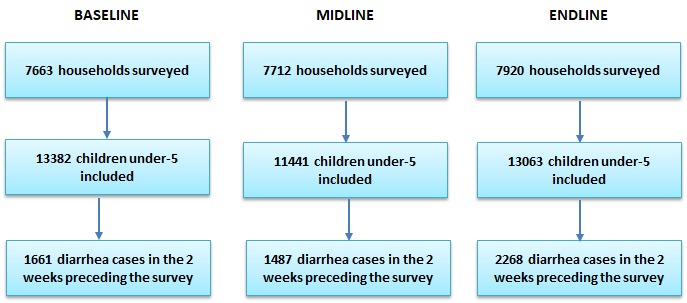
Sample results.

The sampling design aimed to obtain a state-representative sample of households with children under five. For logistical and financial purposes, we performed clustered sampling by randomly selecting households within randomly selected census enumeration areas (EAs). The sampling frame for the study was the 2006 national census. Working with Nigeria’s National Bureau of Statistics (NBS), we first stratified all EAs by state, and urban and rural areas. Next, we randomly selected EAs by using a random number generator in Microsoft Excel (Microsoft Inc, Seattle WA, USA). Population figures were not available for census EAs so we could not conduct probability proportional to size sampling. The sizes of the EAs varied, and we incorporated sampling weights to account for differences in EA sizes which are described later in the paper. Within each EA, trained enumerators listed all households living in the EA and screened for households with at least one child under five living in the household. After completion of the listing, the interviewers conducted a systematic random sample of households with children under five. Based on our experience during piloting, we found that sampling 20 households with at least one child under five per EA was achievable even in the smallest EAs in all states except for Cross Rivers and Rivers where we reduced the number to 15. Due to security issues during data collection, we excluded certain EAs from the sample selection. A new sample was created for each wave of data collection. **Figure 2** presents the number of households interviewed at each wave and the number of children under five and diarrhea episodes included in the sample.

### Data collection procedures

Independent research firms were hired to conduct the data collection: Nielsen Nigeria for the baseline and Practical Sampling International for the midline and endline. The research firms hired enumerators who were familiar with the local context, could speak the local languages in the study states, and had experience conducting household surveys. Both male and female enumerators were hired, though depending on the state context, a greater number of female enumerators were hired to comply with local norms on conducting one-on-one interviews with primarily female caregivers. Enumerators were trained for five days prior to the launch of each data collection period. Training covered background on the project, household listing procedures, review of all survey questions, research ethics, use of the electronic data collection software SurveyCTO (Dobility Inc, Cambridge MA, USA), and a day of field practice. Teams consisting of four interviewers and one supervisor were formed.

The target respondent for the household survey was the primary caregiver of children under five in the household. The household survey included questions regarding household characteristics, respondent characteristics, characteristics of the child, and if the child had diarrhea in the two weeks preceding the survey, detailed information regarding the resolution of the diarrhea, such as source of care and whether the child was given ORS and zinc. Flip charts of local ORS, zinc, and other diarrhea treatments were used to aid the respondent’s recall. The survey was translated into Hausa, Pidgin, and Yoruba, and back translated to English to ensure the question’s intent was not lost. The baseline survey was conducted on paper and double entered into CSPro (US Census Bureau, Washington DC, USA). Discrepant entries were resolved by a third data entrant. The midline and endline surveys were conducted electronically using mobile devices programmed with SurveyCTO.

Data collection was also organized by funding source. Activities in Kano, Lagos, and Rivers, including the program evaluation, were funded by Norad while activities in Bauchi, Cross River, Kaduna, Katsina, and Niger were funded by GAC. Due to differences in donor reporting timelines, data collection for Norad-supported states were different than GAC-supported states. Additionally, the presidential election in 2015 also affected the timing of the Norad midline survey as Nigerians often return to their home state to vote rather than vote in their usual place of residence. The program was also concerned with any potential disruptions that may have resulted from the presidential elections. To avoid effects of population movement during the elections and potential disruptions after the elections, the program decided to conduct the midline prior to the elections. **Table 3** presents the timelines for data collection by funding source.

**Table 3:** Data collection periods

Survey period	Norad-supported program states (Kano, Lagos, Rivers)	GAC-supported program states (Bauchi, Cross River, Kaduna, Katsina, Niger)
Baseline	Dec 2013 – Feb 2014	Sept-Nov 2014
Midline	Mar 2015	Sept-Nov 2015
Endline	Apr-May 2016	May 2017

Norad – Norwegian Agency for Development Cooperation, GAC – Global Affairs Canada

### Sample size calculations

Sample size calculations were designed to ensure adequate power to detect change in ORS and combined ORS and zinc coverage in each state. We used the following formula to calculate the sample size for the study:

where N is the desired sample sizes of children with diarrhea in each state (assuming one child per household), *Deft* is the design effect due to clustering which we assumed to be 1.5 [12], *p1* is the 2011 state coverage estimates (the most recent coverage data available at the time) [11], and *p2* is the endline estimates necessary to see a 25% difference over time. We use a two-sided *t* test with 95% confidence, 80% power, and equal variances. We took into account the prevalence of diarrhea found and allowed for a 5% non-response rate [11]. To simplify training and field work management, assuring better data quality, we used the largest sample size required (ie, Lagos) as the sample size for all states. In each state, the sample size was 940 households with a child under five. Based on the lower density of population within Cross Rivers and Rivers, the sample size was reduced to 930 households with children under five.

### Statistical analyses

We first appended the survey data sets from each state and survey wave into a single data set. We then merged the survey data set with the EA listing data set and constructed probability selection weights and post-stratification weights to account for unequal probability of selection. Additional information on calculations for developing the survey weights is included in Appendix S1 in **Online Supplementary Document**. Descriptive statistics for characteristics of the child, caregiver, and household were calculated for each survey period and were compared using χ-2 tests.

To evaluate whether the five outcomes changed during the program period, we constructed models using generalized estimating equations with the logit link function and computed crude and adjusted odds ratios and 95% confidence intervals. For our fourth and fifth outcomes, we examined if the associations between the survey period and the odds of a child receiving ORS or combined ORS and zinc were modified by source of care by including an interaction between the dummy variables for survey period (ie, baseline, midline, and endline) and source of care (ie, home, public sector, private sector, other sector, or multiple sectors).

For all models above, we identified potential confounders using *a priori* knowledge; these were factors that we believe may be related to use of ORS or combined ORS and zinc as well as factors that may have changed over time. We first conducted bivariate tests between the outcome and each covariate. The final multivariable models included characteristics of the child, caregiver, and household. For child characteristics, we included the child’s sex, child’s age categorized into 1-year age intervals, and source of care for the diarrhea episode. Sources of care were categorized into 5 mutually exclusive categories: did not seek advice or care outside the home, sought care in the public sector only (ie, government hospital or clinic), sought care in the private sector only (ie, private doctor, PPMV, pharmacist), sought care in other source only (ie, market, traditional healer, friend or family), and sought care from multiple different sources. For caregiver characteristics, we included the caregiver’s sex, caregiver’s age categorized into 10-year age intervals, and whether the caregiver had attended any level of schooling. For household characteristics, we included variables for the state the household was located in, urban or rural classification, size of the household, whether the household had access to an improved water source as defined by the Demographic and Health Survey (DHS), and the wealth status. For each survey period and state, wealth scores were independently generated using principle component analysis of survey questions on household assets and categorized into quintiles [13].

To illustrate our model results, we used the multivariable interaction models to estimate predicted probabilities and conducted contrast tests to further evaluate whether there were statistically significant differences in predicted probabilities of treatment by survey period and source of care.

All results incorporated probability selection weights and post-stratification weights to account for unequal probability of selection; additionally, the modeling accounted for the clustering at the census enumeration area and household levels. All analyses were conducted in Stata 14 (StataCorp, College Station TX, USA).

### Ethical approval

The study was reviewed and approved by the National Health Research Ethics Committee of Nigeria (NHREC) (protocol number NHREC/01/01/2007). Ethical approval was also obtained from each state government research ethics committee. Written informed consent was obtained from all participating respondents.

## RESULTS

### Characteristics of caregivers, children, and households

We completed 23 295 interviews resulting in a total of 37 886 children under five listed and a study sample of 5416 children under five with diarrhea in the two weeks preceding the survey (**Figure 2**). The two-week prevalence of diarrhea was found to be 11% at baseline, 13% at midline, and 16% at endline. Prevalence of diarrhea at endline was significantly higher than at baseline (*P* < 0.01). We also found in our descriptive analysis a statistically significant increase in the primary study outcomes. At baseline, we found 38% (95% CI = 34%-42%) of diarrhea episodes received ORS and 4% (95% CI = 3%-5%) received both ORS and zinc. At endline, we found 55% (95% CI = 51%-58%) received ORS and 30% (95% CI = 27%-33%) received both ORS and zinc (**Table 4**).

**Table 4:** Distribution of selected characteristics of children 0-59 mo with diarrhea in the last two weeks by survey, % [95% CI]

Characteristics	Baseline (N = 1661)	Midline (N = 1487)	Endline (N=2268)	*P*-value* (Endline vs Baseline)
**Primary study outcomes**				
ORS treatment	0.38 (0.34, 0.42)	0.54 (0.5, 0.58)	0.55 (0.51, 0.58)	0.000
Combined ORS and zinc treatment	0.04 (0.03, 0.05)	0.23 (0.2, 0.26)	0.30 (0.27, 0.33)	0.000
**Child**				
Female	0.48 (0.45, 0.52)	0.45 (0.42, 0.48)	0.47 (0.45, 0.49)	0.769
Age (months):				
0-11	0.12 (0.1, 0.15)	0.16 (0.13, 0.18)	0.22 (0.2, 0.24)	0.000
12-23	0.30 (0.27, 0.32)	0.29 (0.26, 0.32)	0.32 (0.3, 0.34)	0.170
24-35	0.19 (0.17, 0.22)	0.22 (0.2, 0.25)	0.23 (0.21, 0.25)	0.083
36-47	0.19 (0.17, 0.22)	0.18 (0.16, 0.2)	0.15 (0.13, 0.17)	0.005
48-59	0.20 (0.17, 0.22)	0.15 (0.13, 0.18)	0.09 (0.08, 0.1)	0.000
**Source of care**				
Did not seek care or advice outside the home	0.34 (0.3, 0.38)	0.33 (0.3, 0.36)	0.27 (0.25, 0.3)	0.005
Sought care in public sector	0.25 (0.22, 0.28)	0.29 (0.25, 0.33)	0.27 (0.24, 0.3)	0.436
Sought care in private sector	0.34 (0.3, 0.38)	0.33 (0.3, 0.37)	0.38 (0.35, 0.41)	0.093
Sought care in other place	0.02 (0.01, 0.03)	0.02 (0.01, 0.03)	0.03 (0.02, 0.04)	0.200
Sought care from multiple sectors	0.05 (0.04, 0.07)	0.03 (0.02, 0.04)	0.05 (0.04, 0.07)	0.901
**Respondent/child's caretaker**				
Female	0.90 (0.88, 0.92)	0.93 (0.91, 0.94)	0.94 (0.92, 0.96)	0.013
Age (years):				
15-19	0.04 (0.03, 0.06)	0.03 (0.02, 0.04)	0.03 (0.02, 0.04)	0.027
20-29	0.49 (0.45, 0.53)	0.51 (0.48, 0.55)	0.53 (0.5, 0.56)	0.122
30-39	0.31 (0.27, 0.34)	0.34 (0.3, 0.37)	0.33 (0.3, 0.36)	0.325
40-49	0.11 (0.08, 0.15)	0.09 (0.07, 0.11)	0.08 (0.06, 0.09)	0.035
50-59	0.03 (0.02, 0.05)	0.02 (0.01, 0.03)	0.02 (0.02, 0.04)	0.482
60+	0.02 (0.01, 0.03)	0.02 (0.01, 0.03)	0.01 (0.01, 0.02)	0.310
Attended any level of schooling	0.51 (0.46, 0.55)	0.57 (0.52, 0.62)	0.56 (0.52, 0.61)	0.056
**Household**				
Rural	0.65 (0.6, 0.69)	0.53 (0.48, 0.57)	0.55 (0.51, 0.59)	0.002
**Size of household**				
2-4	0.29 (0.26, 0.33)	0.33 (0.3, 0.37)	0.32 (0.29, 0.35)	0.213
5-7	0.39 (0.35, 0.43)	0.41 (0.38, 0.45)	0.40 (0.38, 0.43)	0.548
8-10	0.18 (0.16, 0.21)	0.14 (0.11, 0.16)	0.16 (0.13, 0.18)	0.104
11+	0.13 (0.11, 0.16)	0.12 (0.09, 0.15)	0.12 (0.1, 0.15)	0.420
Improved water source*	0.48 (0.43, 0.53)	0.50 (0.46, 0.54)	0.51 (0.46, 0.56)	0.384
**State**				
Lagos	0.08 (0.05, 0.12)	0.15 (0.11, 0.19)	0.11 (0.09, 0.13)	0.268
Kano	0.21 (0.17, 0.25)	0.26 (0.23, 0.3)	0.23 (0.2, 0.27)	0.309
Rivers	0.10 (0.08, 0.13)	0.09 (0.07, 0.11)	0.06 (0.04, 0.08)	0.005
Bauchi	0.14 (0.12, 0.18)	0.09 (0.07, 0.11)	0.10 (0.07, 0.13)	0.030
Cross River	0.07 (0.05, 0.09)	0.03 (0.03, 0.04)	0.06 (0.05, 0.07)	0.794
Kaduna	0.14 (0.1, 0.18)	0.09 (0.08, 0.11)	0.16 (0.13, 0.19)	0.399
Katsina	0.14 (0.12, 0.17)	0.16 (0.13, 0.19)	0.16 (0.13, 0.18)	0.454
Niger	0.12 (0.1, 0.15)	0.13 (0.11, 0.16)	0.13 (0.11, 0.15)	0.495
**Wealth**				
Poorest	0.20 (0.17, 0.24)	0.21 (0.18, 0.25)	0.22 (0.19, 0.26)	0.446
Second	0.19 (0.16, 0.23)	0.20 (0.17, 0.24)	0.21 (0.19, 0.24)	0.422
Middle	0.20 (0.16, 0.24)	0.21 (0.18, 0.25)	0.22 (0.19, 0.24)	0.538
Fourth	0.19 (0.16, 0.23)	0.20 (0.17, 0.23)	0.18 (0.16, 0.22)	0.723
Richest	0.21 (0.17, 0.26)	0.17 (0.14, 0.22)	0.17 (0.14, 0.2)	0.092

CI – confidence interval

*P–values were generated comparing baseline and endline values in Stata 14.2 using Pearson χ2 tests

†Improved water source follows the definition used in the Demographic and Health Survey which includes piped water, tube well or borehole, protected well, protected spring, rainwater, and bottled water. Unimproved water source includes unprotected well, unprotected spring, tanker truck/cart, surface water, sachet water, and other sources.

Characteristics of diarrhea episodes in the last two weeks preceding the survey at each time period were generally similar (**Table 4**). There were no statistically significant differences between endline and baseline in the sex of the child with diarrhea, the schooling of the child’s caregiver, and the household size, wealth status, and access to improved drinking water. A few notable exceptions were age of the child, age and sex of the caregiver respondent, and location of the household. At endline, 22% (95% CI = 20%, 24%) of diarrhea episodes were among children 0-11 month compared to 12% (95% CI = 10%, 15%) at baseline. Conversely, there were less children age 36-47 months and 48-59 months at endline than at baseline. Only 15% (95% CI = 13%, 17%) of diarrhea episodes in our sample at endline were among children 36-47 months compared to 19% (95% CI = 17%, 22%) at baseline, and children 48-59 months old made up 9% (95% CI = 8%, 10%) of our sample of diarrhea episodes at endline compared to 20% (95% CI = 17%, 22%) at baseline. We found that more of our caregiver respondents were female at endline compared to baseline. At endline, 94% (95% CI = 92%, 96%) of our caregiver respondents were female compared to 90% (95% CI = 88%, 92%) at baseline. The sample of diarrhea episodes at endline was less rural than our sample at baseline. At endline, 55% (95% CI = 51%, 59%) of diarrhea episodes came from rural areas while at baseline the figure was 65% (95% CI = 60%, 69%). Lastly, a lower proportion of diarrhea episodes came from Rivers state (*P* < 0.01) and a greater proportion of diarrhea episodes were found in Cross River at endline compared to baseline (*P* = 0.03).

### Association between survey period and seeking care for diarrhea

**Table 5** presents crude and adjusted odds ratios (aOR) for our first outcome of seeking advice or care outside the home by survey period. From the multivariable model, the adjusted odds of caregivers seeking care at midline were not significantly greater than at baseline (aOR: 1.16; 95% CI = 0.91, 1.50; *P* = 0.24). At endline, however, the adjusted odds of care-seeking were 1.43 (95% CI = 1.12, 1.82) times greater than baseline (*P* < 0.01).

**Table 5:** Generalized Estimating Equations analyses of the association between survey period and care-seeking among children with diarrhea in the two-weeks preceding the survey (N = 5416)

Covariates	Proportion of children whose caregivers sought care	cOR (95% CI)	aOR* (95% CI)	*P*-value
**Survey period**				
Baseline	0.69 (0.65, 0.72)	Ref	Ref	
Midline	0.68 (0.65, 0.71)	1.04 (0.81, 1.32)	1.16 (0.91, 1.5)	0.235
Endline	0.73 (0.7, 0.76)	1.32 (1.04, 1.68)	1.43 (1.12, 1.82)	0.004

cOR – crude odds ratio, aOR – adjusted odds ratio

*All multivariable analyses adjusted for the above–listed variables and: sex of child; age of child; sex of the caregiver, age of the caregiver, any level of schooling for the caregiver, urban/rural household, size of household, household access to improved water source, state, and household wealth status

### Association between survey period and treatment with ORS and zinc

**Table 6** presents crude and adjusted odds ratios for our primary study outcomes: treatment with ORS and treatment with combined ORS and zinc by survey period and source of care. Adjusting for all other covariates, the overall odds of diarrhea episodes being treated with ORS were 1.80 (95% CI = 1.37, 2.38) times greater at midline than baseline and 1.88 (95% CI = 1.46, 2.43) times greater at endline. The adjusted odds of diarrhea episodes being treated with ORS and zinc combined was 9.63 (95% CI = 6.10, 15.21) times greater at midline and 15.14 (95% CI = 9.82, 23.34) times greater at endline.

**Table 6:** Generalized Estimating Equations analyses of the association between survey period and receipt of ORS and zinc treatment among children with diarrhea in the two-weeks preceding the survey (N = 5416)

Covariates	Outcome: ORS treatment				Outcome: Combined ORS and zinc treatment			
**cOR (95% CI)**	aOR* - no interaction (95% CI)	aOR* - with interaction (95% CI)	**P-value of aOR with interaction**	**cOR (95% CI)**	aOR* - no interaction (95% CI)	aOR* - with interaction (95% CI)	**P-value of aOR with interaction**
**Survey period**								
Baseline	Ref	Ref	Ref		Ref	Ref	Ref	
Midline	1.79 (1.42, 2.26)	1.80 (1.37, 2.38)	1.43 (0.86, 2.35)	0.164	6.52 (4.09, 10.4)	9.63 (6.1, 15.21)	5.93 (2.23, 15.81)	<0.001
Endline	1.92 (1.53, 2.42)	1.88 (1.46, 2.43)	0.89 (0.54, 1.46)	0.634	10.01 (6.34, 15.8)	15.14 (9.82, 23.34)	4.24 (1.59, 11.27)	0.004
**Source of care**								
Did not seek care or advice outside the home	Ref	Ref	Ref		Ref	Ref	Ref	
Sought care in public sector	6.43 (5.09, 8.13)	6.78 (5.21, 8.84)	3.44 (2.04, 5.8)	<0.001	5.79 (4.29, 7.82)	5.78 (4.22, 7.92)	2.64 (0.84, 8.29)	0.097
Sought care in private sector	2.90 (2.37, 3.53)	3.26 (2.6, 4.09)	2.15 (1.26, 3.65)	0.005	2.90 (2.14, 3.94)	2.70 (1.97, 3.69)	0.83 (0.27, 2.57)	0.745
Sought care in other place	0.85 (0.5, 1.45)	0.84 (0.47, 1.48)	0.45 (0.14, 1.46)	0.185	0.75 (0.36, 1.55)	0.61 (0.27, 1.36)	1.50 (0.65, 3.48)	0.340
Sought care from multiple sectors	5.23 (3.59, 7.62)	6.34 (4.01, 10.01)	6.01 (3.13, 11.54)	<0.001	6.45 (4.1, 10.13)	6.36 (3.82, 10.6)	2.18 (0.55, 8.7)	0.268
**Baseline vs endline**								
Did not seek care or advice outside the home			Ref				Ref	
Sought care in public sector			4.49 (2.21, 9.11)	<0.001			3.94 (1.16, 13.41)	0.028
Sought care in private sector			2.43 (1.31, 4.52)	0.005			5.64 (1.67, 19.09)	0.005
Sought care in other place			3.40 (0.84, 13.84)	0.087			1.00 (0, 0)	<0.001
Sought care from multiple sectors			1.38 (0.52, 3.61)	0.518			4.42 (0.94, 20.65)	0.059

ORS – oral rehydration solution, cOR – crude odds ratio, aOR – adjusted odds ratio

*All multivariable analyses adjusted for the above–listed variables and: sex of child; age of child; sex of the caregiver, age of the caregiver, any level of schooling for the caregiver, urban/rural household, size of household, household access to improved water source, state, and household wealth status.

When we included the interaction term to investigate whether the odds ratios between the endline and baseline survey were modified by source of care, we found statistically significant results for treatment of ORS among diarrhea episodes that sought care in the public and private sector. For diarrhea episodes that did not seek care, the adjusted odds ratio of receiving ORS at endline compared to baseline was 0.84 (95% CI = 0.54, 1.46). Relative to this, the odds of receiving ORS is further increased by a factor of 4.49 (95% CI = 2.21, 9.11) when seeking care in the public sector and 2.43 (95% CI = 1.31, 4.52) when seeking care in the private sector. For receipt of combined ORS and zinc, we found the odds ratio for endline was also significantly modified by seeking care in the public sector and private sectors. Among cases not seeking care, the adjusted odds ratio at endline compared to baseline was 4.24 (95% CI = 1.59, 11.27). The adjusted odds ratio was further elevated by a factor of 3.94 (95% CI = 1.16, 13.41) times when seeking care in the public sector and 5.64 (95% CI = 1.67, 19.09) times for cases seeking care in the private sector (*P* < 0.01).

To further illustrate how ORS treatment by survey period is modified by source of care, **Table 7** presents predicted probabilities from the interaction models for treatment with ORS and treatment with ORS and zinc combined by survey period and source of care. Among cases not seeking advice or care outside the home, the predicted probability of receiving ORS was 26% (95% CI = 18%, 33%) at baseline and 24% (95% CI = 19%, 28%) at endline with no statistically significant difference between the survey periods (*P* = 0.64). Among cases seeking care in the public sector, the predicted probability of receiving ORS increased from 57% (95% CI = 50%, 65%) to 83% (95% CI = 79%, 87%) between baseline and endline (*P* < 0.01). The predicted probabilities also increased among cases seeking care in the private sector from 41% (95% CI = 34%, 48%) to 58% (95% CI = 54%, 63%). There was no statistically significant difference in the predicted probability for receiving ORS among cases seeking care from other sources or multiple sectors.

**Table 7:** Predictive probability of children under five with diarrhea in the two weeks preceding the survey receiving ORS and combined ORS and Zinc by source of care

	ORS treatment			Combined ORS and zinc treatment		
**Baseline (95% CI)**	**Endline (95% CI)**	*P*-value*	**Baseline (95% CI)**	**Endline (95% CI)**	*P*-value*
**Endline vs baseline**						
Did not seek care or advice outside the home	0.26 (0.18, 0.33)	0.24 (0.19, 0.28)	0.638	0.02 (0, 0.04)	0.08 (0.06, 0.11)	<0.001
Sought care in public sector	0.57 (0.5, 0.65)	0.83 (0.79, 0.87)	<0.001	0.07 (0.03, 0.11)	0.51 (0.47, 0.56)	<0.001
Sought care in private sector	0.41 (0.34, 0.48)	0.58 (0.54, 0.63)	<0.001	0.02 (0.01, 0.03)	0.29 (0.26, 0.33)	<0.001
Sought care in other place	0.13 (0.01, 0.25)	0.30 (0.16, 0.45)	0.073	†	0.10 (0.03, 0.17)	†
Sought care from multiple sectors	0.64 (0.52, 0.75)	0.68 (0.54, 0.82)	0.652	0.05 (0, 0.1)	0.46 (0.34, 0.57)	<0.001

ORS – oral rehydration solution, CI – confidence interval

*P-values for the change in predictive probability between baseline and endline within each source of care.

†Not estimable due to zero observations within the cell.

For receipt of combined ORS and zinc, there was a statistically significant increase in all sources of care. Among cases not seeking advice or care outside the home, the predicted probability increased from 2% (95% CI = 0%, 4%) to 8% (95% CI = 6%, 11%). Cases seeking care in the public sector saw the largest increase in predicted probability from 7% (95% CI = 3%, 11%) to 51% (95% CI = 47%, 56%). Among cases seeking care in the private sector, the predicted probability increased from 2% (95% CI = 1%, 3%) to 29% (95% CI = 26%, 33%).

## DISCUSSION

Use of ORS and combined ORS and zinc for treatment of diarrhea in children under five significantly increased in focal states during the program period. We also found that the increases in treatment with ORS and combined ORS and zinc treatment were modified by where treatment was sought. The results suggest that overall improvements in ORS and combined ORS and zinc usage was driven by changes in treatment found among diarrhea episodes seeking care in the public and private sector. Use of ORS and combined ORS and zinc improved the most among cases seeking care in these sectors, while diarrhea episodes that did not seek care outside the home or that sought advice or care in other sectors saw no statistically significant change in their probability of receiving ORS and limited increase for combined ORS and zinc. Care-seeking had also increased by endline, likely facilitating improved overall coverage.

The program operated at a large scale and activities were implemented with high intensity. The program reached tens of thousands of health care providers in both the public and private sectors by using multiple channels and forums, such as state-level trainings, supportive supervision, detailing, continuous medical education (CME) meetings, professional associations, and SMS messages. Furthermore, the program had numerous contacts with the providers to reinforce messages. A similar multi-channel, high frequency approach was used to engage with community members. The results of the surveys, taken in context with the activities of the program, suggest that the program likely contributed to the increased uptake of ORS.

This program evaluation is one of the few large-scale program evaluations of ORS and zinc scale-up. Other large-scale program evaluations include the Scaling Up of Zinc for Young Children (SUZY) program in Bangladesh and the Point-of-Use Water Disinfection and Zinc Treatment (POUZN) in Nepal. The SUZY and POUZN programs were among the first national scale-up campaigns of ORS and zinc treatment, and both program designs included government leadership, encouraging local manufacturing, partnerships with private provider associations, and caregiver-targeted demand generation. The SUZY and POUZN programs achieved 20% and 15% coverage of zinc, respectively [14,15]. In Ghana, the Sustaining Health Outcomes through the Private Sector (SHOPS) implemented a program in 3 regions which covered approximately one-third of the country’s population [16]. The SHOPS program used a model similar to SUZY and POUZN and achieved ORS and zinc coverage of 29%. Early successes in the promotion of oral rehydration therapy (ORT) and ORS in the 1980s and 1990s also reference comprehensive interventions addressing policy, supply, provider practice, and community education with large scale activities with that were tailored to the context of the country. In Bangladesh, the organization BRAC went door-to-door and taught over 12 million mothers how to prepare ORS at home. To reinforce and expand the reach of the messages, the Bangladesh program also used school meetings, village healer meetings, print materials, and mass media [17]. Today, Bangladesh has the highest rate of ORS coverage at 77% and combined ORS and zinc use at 38% [18]. Similarly in Egypt, the National Control of Diarrheal Diseases Project (NCDDP) including training of over 16 000 doctors, 8000 nurses, and 320 government supervisors, establishment of ORT corners in 99% of facilities, and mass media campaigns that included TV, print, and radio [19]. Studies of historical efforts have long called for programs to take a comprehensive approach that includes government leadership, partner coordination, the public sector, the private sector, and demand generation [20-23]. This program evaluation further adds to the body of evidence that a comprehensive, locally-tailored program can rapidly achieve increases in ORS and zinc use at scale.

The limitations of the study are that the results are based on a pre-post evaluation design and not a traditional impact evaluation. Thus, we are unable to conclusively attribute changes in use of ORS and zinc treatment to the program. However, the regression models used to describe the associations accounted for multiple sample differences and estimated the most accurate change possible given the design limitations. Additionally, given the nature of the program to support the government to implement its *National Essential Childhood Medicines Scale-up Plan*, several elements of the support were expected to affect all states, thus not allowing for a traditional control comparison. For example, the program helped to strengthen local manufacturing and distribution by providing technical support to source low-cost, high-quality pharmaceutical ingredients and product registration. Sales and distribution data provided by the manufacturers show that their ORS and zinc products were sold widely beyond the program states—and manufacturer and distributor partners actively pursued distribution channels outside focus states during program execution. Thus, a study using other states as comparators may underestimate the impact of the program. Regardless, we do believe this association between the survey period and the odds in treatment is a good representation of program change due to the rigor of the sample and analytical design.

As mentioned in the program description, the program was part of a larger coordinated effort to improve child health outcomes nationally. Other organizations were also collaborating with the government and CHAI through the NEMCM to invest in complementary activities in the program states as well as other states. For example, UNICEF had provided donations of ORS and zinc in Bauchi, Kaduna, and Katsina states at the start of the program. Abt Associates worked in 8 states – overlapping with CHAI in Kano, Kaduna, and Lagos – to train public and private facilities on maternal, neonatal and child health services. Society for Family Health (SFH) conducted mass media radio campaigns in Bauchi, Cross River, Lagos, Kano, and Kaduna that included messages on diarrhea treatment. Thus, the findings likely represent this collective and coordinated effort, and we cannot disentangle the program results from other partner efforts.

Though several activities likely had national influence and other partners were active in some other states, the program activities in the eight states would have likely led to greater coverage than in other states with less intensive activities. We analyzed the UNICEF MICS 2016-17 data and find that combined ORS and zinc coverage in the eight program states were significantly higher than in the rest of Nigeria – 25% (95% CI = 22%, 28%) compared to 14% (95% CI = 12%, 16%) (Table S2 in **Online Supplementary Document**). Again, it is hard to disentangle the effects of the program support to specific states from the market shaping and national-level coordination efforts given the multitude of partners providing support both within the program states and to other states as well. Overall, however, the increased coverage of combined ORS and zinc in both program states and non-program states is suggestive that the program may have had an effect nationally.

The study relied on self-reported information by caregivers of children. We tried to limit bias from this data collection method by limiting our assessment to diarrhea episodes that occurred two weeks prior to the survey. ORS is likely distinguishable from other diarrhea medicines since it is a powder that must be mixed with water. Zinc DT may be confused with other medicines in tablet form. However, to improve the reliability of caregiver reports, data collectors were equipped with flipcharts of locally branded treatments, including ORS, zinc, antibiotics, anti-diarrheals, and home remedies, to aid in recall of the treatment used for the diarrhea episode. We also accepted “don’t know” as a response in case the respondent could not remember the medicine used. The ORS and zinc brands for which the program provided technical assistance to manufacturers are likely not distinguishable to a caregiver from other ORS and zinc brands. However, the goal of the program was not to increase utilization of any specific ORS and zinc brand but to increase overall use of ORS and zinc.

The timing of the surveys could also have affected the results of the survey. As mentioned above, the surveys were timed with donor reporting periods and to avoid any disruptions due to the elections. In the Norad-supported states, the surveys occurred between December to May, while in GAC-supported states, the baseline and midline surveys were conducted between September and November and the endline in May. These months typically have lower rainfall than the summer months of June to August, which may limit any effect of seasonality on treatment rates with ORS and zinc [24,25]. We did find the prevalence of diarrhea was similar between baseline and midline but had increased at endline, likely due to the endline surveys taking place in May and closer to the summer rainy months than the baseline and midline surveys. Although the incidences of diarrhea and diarrheal deaths have clear seasonal variation with incidence typically peaking during rainy months, we found no evidence that treatment with ORS or ORS and zinc would vary by season [26-29]. Surveys such as the DHS and MICS also typically conduct surveys throughout different parts of the year and have been used as benchmarks for measuring change in treatment.

Lastly, we acknowledge that the authors of this study were also involved in the design and implementation of the program. We attempted to mitigate the bias by hiring an external research agency to oversee the data collection. Additionally, we have compared our survey results with independent external data sources. A DHS was conducted in 2013, approximately 6 months prior to the baseline survey [12]. A secondary analysis of the DHS finds that the pooled, weighted coverage for ORS in the eight program states was 39% and combined ORS and zinc was 3% (Table S3 in **Online Supplementary Document**). As shown in **Table 4** we found ORS coverage was 38% at baseline and combined ORS and zinc coverage was 4%. In addition, the UNICEF MICS 2016-17 survey was conducted approximately four months after the endline in Kano, Lagos, and Rivers, but approximately four months prior to the endline in the other five states. The pooled, weighted combined ORS and zinc coverage estimates for the eight program states were 25% (95% CI = 22%-28%) in the MICS 2016-17 results while we found combined ORS and zinc coverage to be 30% (95% CI = 27%-33%) in our endline surveys. As already discussed, our secondary analysis of the MICS 2016-17 survey also found that the combined ORS and zinc coverage in the eight program states was significantly higher than in other Nigerian states. We did find however higher coverage of ORS in our endline survey (55%) than the MICS 2016-17 (40%), which may be due to survey timing, sampling, or how the questions were asked. Our survey was focused exclusively on diarrhea and interviewers carried pictures of local ORS and zinc brands to improve recall, while the MICS survey covers an extensive range of child health and education questions and do not use medicine picture boards to aid recall.

The study limitations notwithstanding, we find that the results taken together with the program context suggest that the program likely contributed to the improvement in ORS and combined ORS and zinc treatment. We encourage evaluations of other national or large-scale programs to further contribute evidence and lessons on designing and implementing large-scale programs. Further rigorous research is also needed to understand what program components and interventions are most effective at driving coverage changes.

## CONCLUSIONS

Use of ORS and combined ORS and zinc for treatment of diarrhea in children under five significantly increased in program states during the program period. Rapid scale-up of ORS and combined ORS and zinc treatment is attainable through a focused, comprehensive diarrhea management program with tailored interventions for the local context.

## Additional material

Online Supplementary Document
