# Supplementary material for: Program evaluation of an ORS and zinc scale-up program in 8 Nigerian states
Source: J Glob Health. 2018 Feb 15;9(1):010502. doi: 10.7189/jogh.09.010502 (PMC6505637; doi:10.7189/jogh.09.010502)
Supplement: Online Supplementary Document [file jogh-09-010502-s001.pdf]

Lam et al. J Glob Health 2019; 9: 010502

Online Supplementary Documents

Lam et al. A program evaluation of an ORS and zinc scale-up program in 8 Nigerian states

Contents

Table S1. Detailed program activities and statistics 2

Appendix S1. Survey weighting 5

Table S2. Combined ORS and zinc coverage by program area in MICS 2016-17 8

Table S3. ORS and combined ORS and zinc coverage in program states in DHS 2013 and MICS 2016-17 8

Table S1. Detailed program activities and statistics

Domain	Between baseline and midline	Between midline and endline
Policy revision and partner coordination	Worked with government to develop a national Essential Childhood Medicines Scale-Up Plan outlining a coordinated strategy for scaling up ORS and zinc use.	Coordinated mass media demand generation activities with BBC Media Action and the Sustaining Health Outcomes through the Private Sector (SHOPS) to reach additional states.
	Established National and State Essential Medicine Coordinating Mechanisms (N/SEMCM) to coordinate domestic and donor resources for implementing the Essential Childhood Medicines Scale-Up Plan.	Coordinated community inter-personal engagements and PPMV outreach with Pact, Society for Family Health (SFH), and SHOPS.
	Supported government to revise the national standard treatment guidelines, national standing orders, and the essential medicines list to include zinc as well as broadly communicate policy allowing zinc to be given over-the-counter.	
	Mapped partner activities and investments to identify opportunities for coordination.	
Market shaping and improving availability of affordable, high-quality ORS and zinc	Facilitated the entry of 4 new low-osmolarity ORS (L-ORS), 5 new zinc DT products, and 4 co-packaged products to the Nigerian market.	Technical support to suppliers to introduce an additional 3 co-packs and 1 zinc DT product.
	Supported local suppliers to reduce wholesale prices by connecting them to more affordable sources of supplies. This led to a 77% reduction in the wholesale cost of a treatment course (2 1L L-ORS sachets & 10 zinc tablets) from \$1.56 to \$0.34	Supported NAFDAC to push existing ORS suppliers to switch to the low-osmolarity formulation.
	Implemented "wholesale activation" to prime the supply chain for ORS and zinc. The activity entailed placing brand-agnostic representatives at regional wholesale hubs to encourage purchase of ORS and zinc and distribute promotional and point-of-sale materials. Reached 5,655 PPMVs.	Conducted 3 additional rounds of wholesaler activation reaching an additional 15,841 PPMVs.
	Signed incentive agreements with local suppliers to implement a rural salesforce. Local suppliers hired and trained 33 rural salesforce team members which sold 273,000 ORS sachets and 288,000 zinc strips.	Continued incentive agreements with local suppliers to implement a rural salesforce resulting in the distribution of 1.7M ORS sachets and 1.6M zinc blister packs to PPMVs and other retailers.
	Provided guidance to state governments on ORS and zinc procurements, including technical assistance with quantification and advocacy to secure budget and funding.	Signed performance-based agreements with nine local distributors to use their existing supply chains to expand distribution of ORS and zinc. Performance based incentives were based on the share of rural PPMV shops carrying ORS and zinc based on independent assessments.
		Continued support to state governments to budget and procure

		ORS and zinc using domestic resources.
Provider training and mentoring	Conducted diarrhea management training sessions with approximately 1,700 leaders from health workers professional associations, such as the Nigerian Medical Association (NMA), Pharmaceutical Society of Nigeria (PSN), National Association of Nigerian Nurses and Midwives (NANNM), and National Association of Proprietary Patent Medicine Vendors (NAPPMED).	Conducted 6 additional rounds of in-facility mentoring sessions resulting in 49,206 repeated mentoring interactions with healthcare workers.
	Conducted 637 state-level training sessions on diarrhea case management. Trainings reached 19,851 public healthcare providers.	Continued one-on-one detailing with PPMVs. Conducted additional 7 rounds of detailing with an additional 82,029 repeated interactions with approximately 30,000 PPMVs reached.
	Supported State Primary Health Care Board (SPHCB) to provide in-facility mentoring to healthcare workers in facilities. Conducted first round reaching 10,928 public healthcare providers.	Imbedded diarrhea management sessions in Continuing Medical Education (CME) sessions and professional association meetings. In total, 331 sessions were conducted reaching approximately 14,000 providers.
	Incorporated ORS and zinc childhood diarrhea management in the pre-service and mandatory training curriculum of PPMVs and Community Pharmacists.	41,235 SMS message sent to public and private providers who had attended trainings to reinforce the training content.
	Collaborated with the state Ministries of Health (MOH), Pharmacists' Council of Nigeria (PCN), and NAPPMED to conduct 589 state-level trainings with PPMVs. Trainings reached 18,670 PPMVs.	
	Began follow-up one-on-one detailing sessions with PPMVs. Conducted 1 round of one-on-one detailing and reached 19,044 PPMVs.	
	17,001 SMS message sent to public and private providers who had attended trainings to reinforce the training content.	
Caregiver demand generation	Developed, tested, and received government approval on messaging and communication materials to promote ORS and zinc to the community.	Adapted caregiver demand generation activities to leverage existing structures, such as female vanguard organizations, religious schools and educational institutions. Over 1,000 sessions discussing children's health and diarrhea were conducted with female vanguard associations and reached over 40,000 women. Over 700 sessions were conducted at Islamiyah schools reaching over 47,000 women.
	Trained 38,825 key community influencers as 'diarrhea champions' to educate caregivers on appropriate diarrhea management and disseminate flyers and other reference materials to share recommendations to their communities.	Expanded "health talks" to over 500 PHCs reaching over 2M caregivers.
	Incorporated diarrhea management content into regular "health	Launched radio campaign in 5 states (Bauchi, Kaduna, Kano,

	talks" at 96 secondary health facilities. "Health talks" targeted mothers visiting antenatal, immunization, and general outpatient clinics. Reached an estimated 448,835 women in the first half of the program.	Katsina, and Rivers). Aired 3,400 radio spots promoting ORS and zinc.

Appendix S1. Survey weighting

Probability selection weights were calculated for each household. We estimated the probability of a household being selected for the survey using the following equation:

$$P_{hi} = P_{1hi} \times P_{2hi}$$

where, P_{hi} is the overall probability of a household in the *ith* EA of stratum *h* being selected, P_{1hi} is the first-stage sampling probability of the *ith* EA in stratum *h* being selected, and P_{2hi} is the second-stage probability of selecting a household in the *ith* EA from stratum *h*.

In the first-stage probability selection, let n_h be the number of EAs selected in stratum *h* and N_h be the total number of EAs in stratum *h* from the sampling frame. The probability of selecting the *ith* EA in stratum *h* is calculated using the following equation:

$$P_{1hi} = \frac{n_h}{N_h}$$

In the second-stage, let g_{hi} be the number of households selected in the *ith* EA, and L_{hi} be the total number of households listed in the *ith* EA of stratum *h*. The probability of a household in the *ith* EA in stratum *h* is calculated using the following equation:

$$P_{2hi} = \frac{g_{hi}}{L_{hi}}$$

Post-stratification weights were also calculated to adjust for non-proportional distribution of states and urban and rural areas. The post-stratification weights aimed to normalize the household sample distribution to approximate the household population distribution found in the 2006 national household census. Post-stratification weights are used to provide estimates that were representative across the entire program area, taking into account population distribution differences between states.

The 2006 national census provides household population estimates for each state. However, the census does not provide information on the number of households living in urban and rural areas. Therefore, we use the proportion of urban and rural EAs from the EA database to estimate the proportion of households living in urban and rural areas. Table 4 summarizes the calculations and estimates. We calculate the proportion of households living in each stratum to the population in the 8 program states.

Estimates of households in each state and by urban/rural areas

STATE	HH POP (A)	URBAN EAS (B)	RURAL EAS (C)	TOTAL EAS (D=B+C)	URBAN EA PROP (E=B/D)	RURAL EA PROP (F=C/D)	URBAN HH POP (G=E*A)	RURAL HH POP (H=F*A)
LAGOS	2,195,842	25,437	0	25,437	1.000	0.000	2,195,842	0
KANO	1,603,335	19,086	17,171	36,257	0.526	0.474	844,009	759,326
RIVERS	1,123,998	10,509	14,362	24,871	0.423	0.577	474,934	649,064
BAUCHI	847,731	3,073	16,839	19,912	0.154	0.846	130,830	716,901
CROSS RIVER	645,251	4,207	11,812	16,019	0.263	0.737	169,459	475,792
KADUNA	1,115,974	12,865	9,226	22,091	0.582	0.418	649,903	466,071
KATSINA	1,066,316	9,781	23,536	33,317	0.294	0.706	313,042	753,274
NIGER	729,964	4,717	19,270	23,987	0.197	0.803	143,546	586,418
TOTAL	9,328,411	89,675	112,216	201,891	0.444	0.556	4,143,450	5,184,961

To calculate the post-stratification weights, we take the ratio between the estimated 2006 household population distribution in the 8 states and our weighted household sample distribution in the dataset. Table 5 summarizes the calculations and the estimates.

Estimates for post-stratification weights

STATE-SECTOR	HH POP (a)	HH POP PROPORTION (B=a/A)	HH WEIGHTED SAMPLE (c)	HH WEIGHTED SAMPLE PROPORTION (D=c/C)	POST-STRATA WEIGHT (B/D)
LAGOS-URBAN	2,195,842	0.235	895,166	0.130	1.807051
KANO-URBAN	844,009	0.090	542,372	0.079	1.146365

KANO-RURAL	759,326	0.081	540,843	0.079	1.034260
RIVERS-URBAN	474,934	0.051	128,024	0.019	2.732847
RIVERS-RURAL	649,064	0.070	150,844	0.022	3.169802
BAUCHI-URBAN	130,830	0.014	143,345	0.021	0.672351
BAUCHI-RURAL	716,901	0.077	848,977	0.124	0.622066
CROSS RIVER-URBAN	169,459	0.018	100,267	0.015	1.245033
CROSS RIVER-RURAL	475,792	0.051	302,288	0.044	1.159495
KADUNA-URBAN	649,903	0.070	626,850	0.091	0.763762
KADUNA-RURAL	466,071	0.050	389,430	0.057	0.881649
KATSINA-URBAN	313,042	0.034	436,233	0.063	0.528637
KATSINA-RURAL	753,274	0.081	1,115,682	0.162	0.497376
NIGER-URBAN	143,546	0.015	113,637	0.017	0.930560
NIGER-RURAL	586,418	0.063	538,002	0.078	0.802964
TOTAL	9,328,411	1.000	6,871,960	1.000	1.000000

Table S2. Combined ORS and zinc coverage by program area in MICS 2016-17

Outcome / Program area	Estimate	SE	95% LB	95% UB	N
Combined ORS and zinc					
Program states*	0.251066	0.015797	0.2213619	0.283305	1,413
Non-program states†	0.141133	0.010934	0.1210142	0.163973	2,361

*Program states are Bauchi, Cross River, Kaduna, Kano, Katsina, Lagos, Niger, Rivers
†Non-program states are the all other states in Nigeria that do not include the program states

Table S3. ORS and combined ORS and zinc coverage in program states in DHS 2013 and MICS 2016-17

Survey / Outcome	Estimate	SE	95% LB	95% UB
DHS 2013 (N=875)				
ORS	0.390721	0.024496	0.3435665	0.440008
Combined ORS and zinc	0.025478	0.007888	0.0137701	0.046669
MICS 2016-17 (N=1,413)				
ORS	0.403291	0.017583	0.3692538	0.438286
Combined ORS and zinc	0.251066	0.015797	0.2213	0.283378

DHS – Demographic and Health Survey, MICS – Multiple Indicator Cluster Survey, ORS – oral rehydration solution
